# Phonological Interlopers Tend to Repeat When Tip-of-the-Tongue States Repeat

**DOI:** 10.3389/fpsyg.2019.00341

**Published:** 2019-02-21

**Authors:** L. Kathleen Oliver, Karin R. Humphreys

**Affiliations:** ^1^Research and Analytics Department, Hamilton-Wentworth District School Board, Hamilton, ON, Canada; ^2^Department of Psychology, Neuroscience & Behaviour, McMaster University, Hamilton, ON, Canada

**Keywords:** tip-of-the-tongue, word production, interlopers, error repetition, error learning

## Abstract

We elicited tip-of-the-tongue (TOT) states to replicate the finding that TOTs repeat for individual words. Humphreys and colleagues have attributed this error repetition phenomenon to implicit learning of the mappings between the lemma and phonology. We also examined whether or not interlopers – repeated information that persistently comes to mind – repeat during TOT states for individual words, along with the type of interlopers. Participants were given a TOT test and the same TOT test one week later. The test consisted of the presentation of a definition and participants indicated if they knew the word, did not know the word, or if they were in a TOT state. Participants were also given 15 s to think aloud about the target word on both test and retest. We found that information repeats significantly more often for repeated TOT states (26%) than repeated Don’t Know states (13%). We also found that participants experienced significantly more repeated phonological interlopers during a repeated TOT state (59%) versus a repeated Don’t Know state (12%). Theoretically, the results may suggest that the TOT state is best described as a subthreshold state, and that within this subthreshold state there is a specific erroneous pattern of activation (akin to a local minimum) rather than a non-specific pattern of activation. These findings are an important constraint toward the development of a more formal explanation of recurring TOTs.

## Introduction

A tip-of-the-tongue (TOT) state is the feeling of knowing that one knows a word, yet being unable to say it (Brown and McNeill, 1966). The experience of being in a TOT state is typically frustrating, followed by a feeling of relief if the correct word is attained (Brown, 1991). The TOT state is a unique, universal state that plagues every speaker at least some of the time. The TOT phenomenon is of particular interest to psycholinguists. TOT states are hypothesized to be a direct consequence of a word production failure, which has led to significant and influential findings in the field of word production (e.g., Burke et al., 1991). Burke and colleagues argue that TOT states are the direct consequence of a phonological failure (mechanism explained below). It can be especially frustrating when a TOT state seems to happen repeatedly for the same word, which is a relatively underexplored feature of TOT states; that is, TOT states tend to repeat (Warriner and Humphreys, 2008; D’Angelo and Humphreys, 2015). Humphreys and colleagues argue that TOT states recur due to an error learning mechanism within the word production system. The current paper attempts to replicate the finding that TOT states tend to repeat, but we also explored an additional line of inquiry. Specifically, what is being learned during a TOT state?

### TOT States and the Two-Stage Model of Word Retrieval

The most widely agreed-upon model of language production is a two-stage model, where activation proceeds from a concept – the non-linguistic stage – to the lemma, and the lemma to phonology (Dell, 1986; Badecker et al., 1995; Dell et al., 1997; Levelt et al., 1999). The lemma is defined as an abstract pointer to the target word in that it is a lexical unit that holds syntactic information, but is not yet connected to sound. The lemma is then mapped onto phonemes, which creates the sound of the word. Within this framework, a TOT state is thought to represent some kind of failure of activation in this mapping process, and is specifically hypothesized to reflect a successful retrieval of a lemma, but an unsuccessful subsequent activation of the full phonological representation of that word (Dell, 1986; Burke et al., 1991; Dell et al., 1997; Levelt et al., 1999; Gollan and Brown, 2006; Warriner and Humphreys, 2008; Pureza et al., 2013, 2016; D’Angelo and Humphreys, 2015). A TOT state may occur the first time when phonological failure occurs due to noise in a spreading-activation-like system, which is heightened by relatively weak connections within the word production system (e.g., Dell, 1986; Dell et al., 1997). For example, if a word has not been used for a long period of time or if a word is used on rare occasions, lemma-to-phonology connections may become weakened due to disuse, leading to a TOT state (e.g., Burke et al., 1991). Event-related potential studies have supported the lemma-to-phonology failure account of TOT states (e.g., Díaz et al., 2007).

### Repeated TOT States

Providing some theoretical extensions and modifications to Burke and colleagues’ work, Humphreys and colleagues have shown that TOT states tend to repeat for individual words (Warriner and Humphreys, 2008; D’Angelo and Humphreys, 2015). That is, speakers who experience a tip-of-the-tongue state on a word are more likely to TOT on the same word again than would be predicted by chance; this phenomenon is called the *error repetition effect*. Although a robust error repetition effect has been found, there is uncertainty surrounding the factors that may contribute to this effect. Humphreys and colleagues have argued that this error repetition effect is not simply due to the fact that some words tend to be idiosyncratically difficult for speakers (due to low frequency, low neighborhood density, etc.). Instead, they argue that speakers are learning the error itself. The study of repeated TOT states is relatively distinctive from other TOT research. We are interested in observing what occurs after a speaker enters a TOT state rather than the factors that lead the speaker to enter a TOT state the first time.

Humphreys and colleagues have provided extensive evidence for an error repetition effect as opposed to individual difficulty for a word (Warriner and Humphreys, 2008; D’Angelo and Humphreys, 2015). The first critical piece of evidence for an error learning effect is called the timing manipulation effect. Specifically, learning can be manipulated depending on the amount of time a speaker is given to ruminate on a word. If a repeated TOT state were due to idiosyncratic difficulty, a speaker should be no more likely to repeat a TOT state in the longer time manipulation than the shorter time manipulation. TOT states are more likely to repeat for those who are given more time to think about a target word. The second critical piece of evidence for an error learning effect is called the resolution effect. A speaker is less likely to repeat a TOT state if that particular TOT state was self-resolved the first time. The resolution effect indicates the presence of a self-corrective learning mechanism. The third critical piece of evidence is the phonological cueing effect. When a speaker is given a phonological cue during a TOT state, leading to a subsequent self-resolution, the speaker is less likely to enter that same TOT state again. This same effect is not present when speakers are presented with semantic cues. The phonological cueing effect indicates that word production is interrupted at the lemma-to-phonological stage, and that providing a cue pushes the speaker to activation threshold. By reaching the threshold for activation, the speaker has now activated and strengthened the correct pathway, making another TOT event less likely. This can be described as the language production system learning from experience in that it is constantly being updated with every word spoken (Warriner and Humphreys, 2008; Oppenheim et al., 2010). Theoretically, the TOT state may consist of an incorrect mapping between lemma and phonology, and that incorrect pathway is reinforced via a Hebbian-type learning mechanism, thus making the error more likely in a subsequent retrieval attempt (Warriner and Humphreys, 2008; D’Angelo and Humphreys, 2015).

We attempted to build upon D’Angelo and Humphreys (2015) and Warriner and Humphreys (2008) findings that provide extensive experimental support for an error learning mechanism underlying at least a portion of the error repetition effect. In this paper, we sought to provide an important extension to the findings of Humphreys and colleagues, which is to look into the nature of what is being learned.

### Interlopers

A notable feature of TOT states is that speakers can often recall features of words that are on the tip-of-the-tongue (e.g., Brown and McNeill, 1966; Brown, 1991; Meyer and Bock, 1992; Schwartz and Metcalfe, 2011). These are sometimes referred to as interlopers. These interlopers can be entire words – for example only being able to recall the word “oblong” when trying to recall “obsidian,” or there might be partial phonological information, such as the onset phoneme, or the number of syllables. Related semantic information may also come to mind. This paper looks at interlopers in the context of the error repetition effect in TOT states. When TOTs recur, do corresponding interlopers recur as well? Furthermore, what kinds of interlopers are associated with recurring TOTs? Critically, this information can inform us about the nature of the TOT state itself.

Experimental paradigms that have been used to explore interlopers typically involve the experimenter creating the interloper for the participant (e.g., Maylor, 1990). Interlopers have also been studied through participants self-recording their TOTs and what words come to mind throughout a period of time (e.g., Burke et al., 1991), but thus far there has been no study of experimentally elicited TOT states and naturally occurring interlopers across multiple testing sessions. Interlopers are often presented before TOT elicitation, and although this provides insight as to what happens before a TOT state occurs, by examining naturally occurring interlopers in a controlled setting we can examine what is happening during the TOT state.

### Theoretical Account of TOT States as a Specific State

While Humphreys and colleagues have argued that repeated TOTs represent an error learning effect, the question remains: what is being learned? Previously, it was argued that it might represent some kind of Hebbian learning mechanism, strengthening the connection between the lemma and an incorrect phonological representation, which makes speakers less likely to be able to use the correct mapping on a subsequent retrieval attempt. This bears directly on the question of what the pattern of activation during a TOT state is like. We make the assumption that the lemma has been correctly retrieved, and the TOT state is a function of failure to retrieve a fully correct phonological pattern. The first possibility is that there is a general subthreshold state in which no phonological pattern reaches threshold for selection, although there might be partial or related information retrievable (e.g., Burke et al., 1991). The other possibility is a blocking hypothesis (e.g., Jones, 1989), in which the phonological pattern corresponding to an incorrect word is selected and this prevents the selection of the correct pattern.

Within the idea of the general subthreshold state, however, it is not clear what that state would look like. The frequent ability of speakers to identify partial phonological information, or report sound-alike interloper words certainly suggests a fairly organized pattern of phonological activation that is consciously accessible. We acknowledge that there are cases when participants do not identify partial phonological information. These alternative cases may occur for multiple reasons. First, some participants may be reluctant to report information about the target word for fear of being wrong. Other participants may have certain traits that inhibit them from speaking aloud (e.g., shyness). Our methodology includes having an experimenter in the room to encourage participants to speak aloud. Therefore, it is possible that some participants are reluctant to speak in front of a stranger. In other cases, we speculate that there is enough random noise within the word production system that makes it difficult for a speaker to pinpoint specific phonological information to report. However, we acknowledge that the repetition of a specific TOT state in conjunction with specific phonological information is a statistical tendency and not a systematic one, and at this stage of the development of the error repetition hypothesis, this explanation is more so speculative than it is definitive, but is nonetheless a plausible and promising hypothesis. While non-lexical factors may sometimes play a role in TOT states, we maintain our argument that TOT states are the result of a lemma-to-phonological mapping failure, and that repeated TOT states are the result of learning the erroneous lemma-to-phonological mapping failure.

One possible analogy is that of a local minimum in an autoassociative type network, in which the system has converged on a partial, but ultimately incorrect pattern, and remains trapped in this erroneous state. Alternatively, the subthreshold state might not be associated with a convergence at a local minimum, but remains a state in which simply nothing is activated highly enough to be selected. It is certainly possible that individual TOT instances may sometimes be more like one of these alternatives than the other – i.e., in cases where partial report of an interloper is available, this might represent convergence at a local minimum. In cases where no phonological information is available, there might simply not be enough activation available to form any kind of coherent pattern of activation.

In thinking about possible mechanisms that underlie a learning effect in TOTs, understanding the nature of the underlying state is critical. If in fact TOTs often represent something akin to an erroneous convergence on a local minimum, then the error learning effect can be fairly straightforwardly described as the strengthening of the connection between lemma and that specific erroneous state of phonological activation. In this explanation, that new reinforced mapping is then at an increased likelihood of being used again on a subsequent retrieval, and leading to another TOT. This explanation then makes the prediction that repeated TOTs are likely to have similarly repeated erroneous phonological information available. That is, not only is the failure to retrieve a word repeated, but the specific error state is repeated as well.

### Current Study

This study was designed to investigate error learning in TOT states by exploring the role of interlopers. Data were analyzed to determine if errors repeat, and if interlopers repeat along with them. It was hypothesized that the likelihood of being in a TOT state for a particular word on Day Two will be greater given that a participant was in a TOT state for the same word on Day One, especially for TOTs that were not resolved on the first day, replicating earlier findings. The think-aloud protocols were analyzed to determine whether participants were reporting interlopers, what kind of interlopers they were (e.g., semantic or phonological) and to what extent those interlopers repeated at the subsequent retrieval attempt. If specific interloper information repeats, this is evidence for a TOT state as being a relatively specific erroneous pattern of activation that can be learned, and can reoccur, rather than a non-specific subthreshold activation state. Showing that a specific state of activation occurs during the TOT provides further evidence for a mechanism by which TOT error learning can occur.

## Materials and Methods

### Participants

Participants included 40 native English speaking undergraduate students (36 females, 4 males) from McMaster University. Participants had a mean age of 19.6 years. Twenty-one participants identified as bilingual, but all were recruited as being native English speakers. Ethics approval was received from the McMaster University Ethics Board, and signed consent was obtained from all participants. Course credit was given in exchange for participation. All subjects gave written informed consent in accordance with the Declaration of Helsinki.

## Experimental Measures

### Materials

Participants completed two testing sessions. They were given the same TOT test in both sessions. The test consisted of 80 definitions (Warriner and Humphreys, 2008). There were 70 critical definitions for low frequency words, which were designed to elicit a TOT state. See the Appendix Table A1 for frequency values of whole words, orthographic length, count of phonological neighbors, and mean concreteness ratings. Whole word frequency was obtained from the Corpus of Contemporary American English (Davies, 2008). The majority of counts of phonological neighbors were obtained from The Irvine Phonotactic Online Dictionary, Version 2.0 (Vaden et al., 2009). In cases where words were not available in the database, the Similarity Neighborhood Calculator was used to obtain estimates of phonological neighborhood density (Vitevitch, 2019). Note that there was no specific phonological neighborhood density information for the words “spelunker” or “decanter.” Therefore phonological neighbors for the words “spelunking” and “decanting” were used as approximations for spelunker and decanter. Words may have slightly different counts of phonological neighbors depending on pronunciation. Therefore, the authors chose the pronunciations that were most consistent within the Canadian-English language. Concreteness ratings were derived from Brysbaert et al. (2014). Concreteness was rated on a five-point scale, with a larger number meaning greater concreteness. For some target words, lexical information was not available. There were also 10 fake definitions. Participants were told there were fake definitions, which had no answer. The addition of fake definitions was to ensure that participants were in a true TOT state, by encouraging participants to report when they did not know an answer.

### Procedure

On Day 1, the experimenter explained to participants that they would see definitions of words, and each time they saw a definition they would be asked if they knew the answer, did not know the answer, or if they were in a TOT state. They were told “a tip-of-the-tongue state is the feeling of knowing you know a word, but you are unable to say it.” Participants were also instructed and encouraged to speak aloud during Don’t Know and TOT trials. If participants could not think of the target word, they were told to verbally report any accessible information about the word, e.g., the first letter of the word, number of syllables, or any words that came to mind.

The definitions were visually displayed on a 19-inch ViewSonic Professional series P95f+ CRT color monitor controlled by a Dell Dimension 4600 computer. Spoken responses were recorded electronically via a hand held microphone. Stimuli were presented, and key-press responses recorded, using Presentation^®^ (v.13, neurobs.com) experimental software. The experiment began with written instructions presented on the screen, which included the definition of a TOT state.

#### TOT Test 1

**FIGURE 1:**
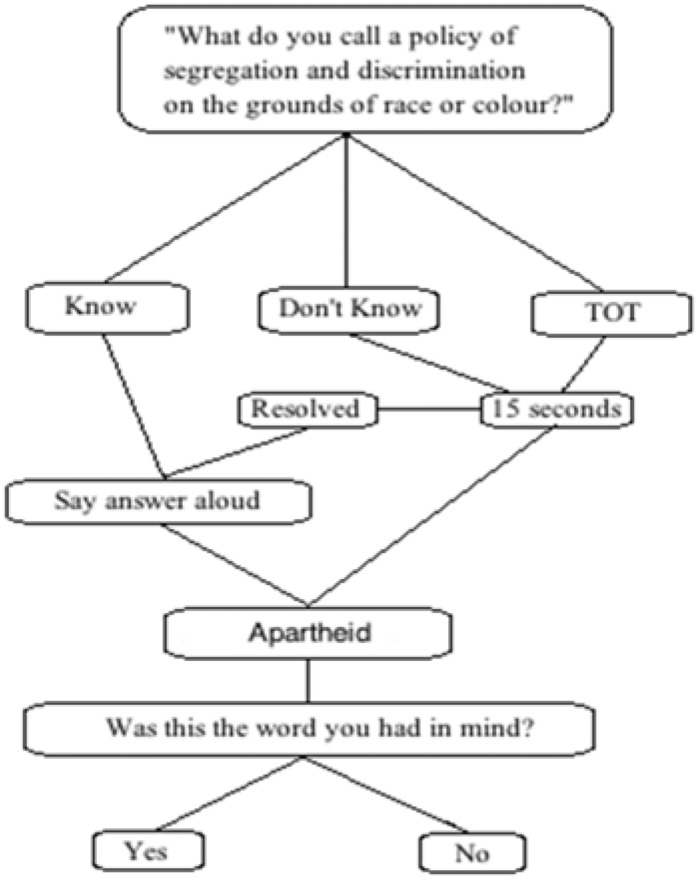
Schematic of a trial used on TOT Test 1 and 2.

Each trial began with the visual presentation of a definition. The keyboard buttons were labeled “Know,” “Don’t Know,” and “TOT.” Participants pressed the button that corresponded with their response. Participants spoke aloud about the target words when in a “TOT” or “Don’t Know” state throughout the entirety of the experiment. See Figure 1 for a schematic of the experimental procedure.

All participants had a 15 s delay period when the response was “Don’t Know” or “TOT.” The definition of the target word remained on the screen for the 15 s. If the participants resolved their TOT during the 15-s delay, they were instructed to press the “Know” button. Participants were then prompted to say the word aloud. If the participants correctly produced the word during the 15-s delay after pressing the “Know” button, this was considered to be a Resolved TOT or Resolved Don’t Know response. While resolved Don’t Know responses may actually be resolved TOT responses, it is impossible for us discern whether or not this is the case. Rather, the aim of giving participants the opportunity to resolve Don’t Know responses was to demonstrate that the resolution of a Don’t Know response rarely occurs in comparison to resolved TOT responses. If participants could not produce the target word by the time the delay period ended, these trials were labeled unresolved. Participants were shown the correct answer at the end of each trial and were asked if that was the word that had been thinking of. If the definition was a fake, it was revealed to participants that there was no corresponding word to the definition. We determined that 15 s was long enough to reinforce the erroneous pathway without having long enough to self-resolve too many trials.

While theoretically, participants should not self-resolve after a “Don’t Know” response, we do present participants with the option to resolve a “Don’t Know” response for two reasons. One is to demonstrate that it is rare for Don’t Know responses to be resolved at all, and second, to demonstrate that those who respond “Don’t Know” are more likely to report semantic information which is indicative of access to the first stage of word production, but not the lemma-to-phonology stage of production. With this information, we can conclude that “Don’t Know” and “TOT” are distinct responses.

#### TOT Test 2

The second TOT test was identical to the first TOT test. The second testing session was one week after the first. Definitions were randomized in both testing sessions. An experimenter was present for both testing sessions and actively encouraged participants to speak aloud. Each session lasted approximately 45 min.

## Results

### Overall Response Likelihoods

If a participant indicated that they knew a word or if a word was on the tip of their tongue and that the target word is the word they were thinking of, we can conclude that this is a valid Know or TOT trial. If they indicated that they did not know a word and that the target word was not the word they were thinking of, we classified this response as a valid Don’t Know.

If a participant indicated that they knew a word or if a word was on the tip of their tongue and that the target word was not the word they were thinking of, we can conclude that this is an invalid Know or TOT trial. If they indicated that they did not know a word and that the target word was indeed the word they were thinking of, we classified this response as an invalid Don’t Know trial. By differentiating between valid and invalid trials we can be sure that our participants are experiencing a true Know, Don’t Know, or TOT state.

This experiment has a total of 2800 pairs of trials collected from 40 participants for 70 definitions across two testing sessions. A trial pair contains the Test 1 response and Test 2 response for an individual target word. Of the 2800 Test 1 trials, 2058 were valid and of the 2800 Test 2 trials, 2296 were valid. This means that 1019 pairs of trials were excluded on the basis that they were invalid. Of all Test 1 Know responses, 26% (308/1175) were invalid, whereas 11% (169/1577) of Test 2 Know responses were invalid. Out of all Don’t Know responses, 14% (139/1018) of Test 1 responses and 16% (115/708) of Test 2 responses were invalid. Of all TOT responses 48% (292/604) of Test 1 TOT responses and 42% (216/511) were invalid. See Table 1 for the valid response rates.

**Table 1:** Overall valid response rates.

Test 1 Responses	Proportion	Test 2 Responses	Proportion
Know	0.42	Know	0.61
Don’t Know	0.43	Don’t Know	0.26
TOT	0.15	TOT	0.13


**FIGURE 2:**
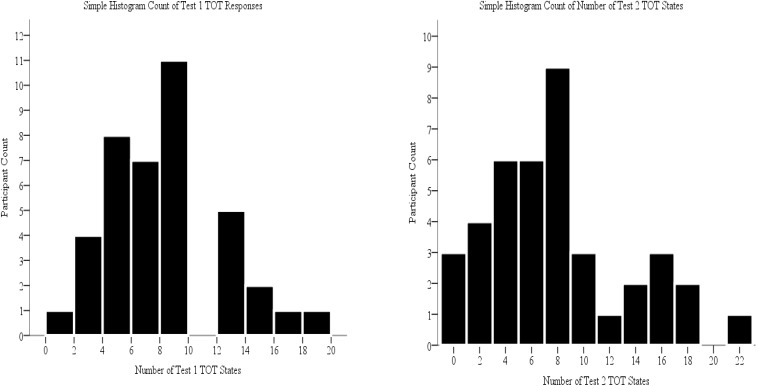
Count of Number of Test 1 TOT Responses and Test 2 TOT Responses.

All 40 participants experienced at least one TOT state on Test 1 (see Figure 2). The number of TOTs per participant ranged between one and nineteen (out of seventy critical trials). Considering that we analyze our data by items, it is important to discern whether or not only a small subset of participants are contributing to the overall TOT rate. Since all participants experienced a TOT state, we can conclude that this is not the case. Thirty-seven out of 40 participants experienced at least one TOT state on Test 2 (see Figure 2).

### Repeated TOT States

We looked at the tendency for TOT states to repeat for individual words on Test 1 and one week later on Test 2. Twenty-eight out of 40 participants experienced at least one repeated TOT state. We also looked at resolved and unresolved TOT states on both Test 1 and Test 2. After collapsing across resolved and unresolved responses, we found that 27% (84/312) of the TOT states experienced on Test 1 were repeated on Test 2.

A log odds ratio statistic was calculated to examine the difference between conditional probabilities of Test 2 response given a Day 1 response. The odds ratio (OR) in this case is the odds of experiencing a TOT on Test 2 versus a Know or Don’t Know response given a TOT or a non-TOT for an individual word on Test 1. The odds ratios were calculated by creating a dichotomous variable that differentiated between a TOT and non-TOT response, which was composed of Know and Don’t Know responses. If an OR is equal to 1 this shows that there is no increased likelihood of having a TOT on Test 2 given that there was a TOT on Test 1. The OR for Test 2 TOTs given a Test 1 TOT response versus non-TOT response was 4.84 with the lower and upper limits of a 95% confidence interval of 3.53 and 6.64 (*z* = 9.79, *p*< 0.01). Note we report the OR rather than log OR, for readability purposes. The OR of 4.84 means that the odds of repeating a TOT on Test 2 were almost five times greater if one experienced a TOT on Test 1 rather than a non-TOT response. These statistics mean that TOT states repeat for individual words at a rate greater than chance than all other responses.

Considering only TOT states that were not spontaneously resolved, unresolved TOTs repeated at a rate greater than chance (OR = 7.29, 5.11 – 10.42, *z* = 10.94, *p* < 0.01). This means that the odds of a TOT repeating on Test 2 for an individual word was over seven times greater if an unresolved TOT was experienced on Test 1 as compared to all other responses.

Next, we looked at the effect of self-resolution of TOT states during the trial. Consistent with the “resolution effect” described by D’Angelo and Humphreys (2015), the probability of experiencing a TOT on Test 2 given that a TOT was left unresolved on Test 1 is 0.41 in comparison to a resolved TOT, which is only 0.15. This means the recurrence of a TOT depends on whether or not a TOT state is resolved on Test 1. The odds of a TOT state repeating on Test 2 for an individual word is almost four times greater if there was an unresolved TOT on Test 1 as compared to a resolved TOT, i.e., successfully finding the answer on one’s own (OR = 3.98, 2.18 – 7.26, *z* = 4.51, *p* < 0.01). See Table 2 for the odds ratio for the difference between resolved and unresolved Test 1 TOTs and conditional probabilities that were calculated for Test 2 responses given a Test 1 response. Given that there were only two resolved Don’t Know trials, these two trials were not included in Table 2.

**Table 2:** Cross-tabulation of Test 1 and Test 2 responses.

	Test 2 Responses					
Test 1 Responses	Know	Don’t Know	TOT	Total	Conditional Probability*^*a*^*	Odds Ratio
Know	815	5	32	852	0.04	
Don’t Know	142	423	90	655	0.14	
TOT Resolved	98	1	17	116	0.15	
TOT Unresolved	86	12	67	165	0.41	3.98^∗^


*^*a*^Conditional probability of experiencing a TOT on Test 2. The odds ratio compares the likelihood of a resolved TOT to repeat versus an unresolved TOT to repeat. Odds ratios are calculated from a dichotic variable of Test 2 TOT versus another Test 2 response. ^∗^*p* < 0.01.*

It may initially appear that resolved TOT states and Don’t Know responses were behaving similarly across test and retest. The conditional probabilities of repeating a TOT state given a Don’t Know or resolved TOT response on Test 1 were similar (0.14 and 0.15, respectively). However, the result is actually supportive of our hypothesis. We expected that both resolved TOT states and Don’t Know responses would be less likely to result in a TOT state at retest. While the conditional probabilities are similar, we hypothesize that Don’t Know and resolved TOT responses are less likely to result in a TOT state on Test 2 for different reasons. When the two-stage model of word production is taken into consideration, a Don’t know response on Test 1 should be less likely to turn into a TOT on Test 2 due to the word being so weakly represented in the lexicon, or perhaps not being present in the lexicon at all. Resolved TOT states on Test 1 may be less likely to turn into TOT states on Test 2 because of the hypothesized self-corrective learning mechanism supported by Humphreys and colleagues.

### Interloper Coding Scheme

Utterances were first transcribed and then coded as semantic or phonological. An utterance is considered to be semantic if the speaker produces a word that is related in meaning to the target word. We also coded utterances from episodic memory as semantic. An utterance is considered to be phonological if the speaker produces an utterance that has an alternative word that is related to the sound of the word. Also within the phonological category is partial phonological information, onset cluster, first syllable, first and second syllable, and orthographic utterances. All other responses were coded as unrelated. Semantic utterances occurred when a participant uttered a word that was related in meaning to the target word, e.g., “nectar” from the target word pollen. Partial sounds derived from the target word were coded as partially phonological, e.g., “something scope” from kaleidoscope. An onset cluster utterance occurred when the speaker was able to think of the onset of consonants in which a vowel is absent, e.g., “ch” from chalice. An example of a first syllable is “pro” from procrastinate and an example of first + second syllable is “aba” from abacus. An orthographic utterance occurred when the speaker was able to provide information about the written form of the word, e.g., “It starts with an “a”’ for the target word arson. An utterance coded as “episodic memory” means the speaker was able to remember information related to the target word such as a time, place, or context in which they encountered the target word, e.g., “It was in the Hiroshima book I read about the Japanese girl” for the target word origami. An example of an unrelated utterance is “billboard” for planetarium. We collapsed across semantic and episodic memory as semantic interlopers, and collapsed across phonological, partial phonological, onset cluster, first syllable, first and second syllable, and orthographic utterances as phonological interlopers. We note that in some cases a TOT state may be elicited from a trial definition for a word that is not the target word. In these cases we may not know the word the participant is thinking of, therefore making it impossible to know whether or not the phonological information provided is related or not.

### Repeated Interlopers

We looked at the tendency for interlopers – persistent information that comes to mind – to repeat for individual words. Participants were given 15 s to think aloud about the target word during a Don’t Know or TOT state. The mean length per utterance was 14.5 words. We calculated the proportion of trials with repeated information on Test 2. Only paired trials that had the same response on Test 1 and Test 2 are reported here, i.e., Don’t Know Test 1/Don’t Know Test 2 for an individual target word and TOT Test 1/TOT Test 2 for an individual target word. Participants were not given 15 s to think aloud if they knew the word, so Know responses are excluded from this analysis. The proportion of paired trials with repeated information was larger for TOT trials than Don’t Know trials. Proportion of trials with repeated information was calculated by dividing the number of trials with the exact same repeated information uttered by the total number of TOT or Don’t Know trials that were repeated on Test 2. The proportion of recurring TOT trial pairs with repeated information was 26% (22/84). In contrast the proportion of Don’t Know trials with repeated information was 13% (57/423) (OR = 2.28, 1.30 – 3.99, *z* = 2.88, *p* = 0.004).

### Interloper Type

Participants reported phonological information 7% (63/879) of the time for Test 1 Don’t Know trials and 34% (105/312) of the time for Test 1 TOT trials (OR = 6.57, 4.64 – 9.30, *z* = 10.61, *p <*0.01). We also looked at the nature of the information that was being repeated; repeated interlopers were coded as phonological or semantic. There were 50 paired Don’t Know trials that included only repeated semantic information, 2 trials that included only repeated phonological information, and 5 trials that included both repeated semantic and phonological information.

The paired TOT trials included 9 trials that included repeated semantic information and 12 trials that included repeated phonological information, and one trial that included both semantic and phonological information (see Table 3 for the 13 repeated TOT trials with repeated phonological interlopers). We note that two of the paired TOT trials produced incorrect phonological (javelin and ornithology). We speculate that although repeated phonological interlopers are not always correct, there is sometimes some random noise within the word production system that leads a speaker down a specific erroneous phonological pathway. We know that participants were indeed in a TOT state for these words as we asked them at the end of each trial if the target word is the word they had in mind, to which the participants said yes.

**Table 3:** Phonological interlopers for repeated TOT responses.

Target	Interloper
abdicate	a
ornithology	o
slalom	s
metronome	m, me
javelin	s
ellipsis	e
ornithology	a
onomatopoeia	o
kaleidoscope	something scope
planetarium	planetorium
pseudonym	s
odometer	speedometer
vineyard	v


Out of the 22 paired TOT trials, 7 trials included a repeated alternative word that was semantically related to the target word. Two trials with repeated semantic information consisted of information from episodic memory, e.g., “There’s one here at McMaster University” for the target word “planetarium.” See Table 4 for the number of interloper types for repeated Don’t Know and TOT trials.

**Table 4:** Count of interloper type for DK and TOT responses.

	Interloper Type						
Trial Pair Response	Alternative Semantic Word	Episodic Memory	Partial Phonology	Orthographic	Alternative Phonological Word	Alternative Semantic + Phonological Word	Total
Don’t Know	48	2	1	1	0	5	57
TOT	7	2	1	10	1	1	22


It is important to note that the critical finding here is not how large the number of trials is, but the difference between semantic and phonological information for Don’t Know trials in comparison to the difference between semantic and phonological trials for unresolved TOTs. Participants reported phonological information on 12% (7/57) of paired Don’t Know trials with repeated information and on 59% (13/22) of paired TOT trials with repeated information (OR = 10.21, 3.27 – 31.92, *z* = 3.99, *p* < 0.01). The likelihood of phonological interlopers repeating could be due to chance. However, due to the relatively small sample size, the likelihood of having specific phonological information repeat becomes vanishingly small.

Note that the difference between semantic and phonological information type is much smaller for the unresolved TOT responses. If one is in an unresolved TOT state they will experience phonological interlopers more than if they were in a Don’t Know state. It is also important to note that the majority of trials with repeated information for the Don’t Know trials include alternative words, whereas the majority of TOT trials with repeated information include partial phonological information directly related to the target word. There was also only one Don’t Know trial that included correct partial phonological information related to the target word.

## Discussion

### Central Findings

The results show that not only do errors repeat one week later for individual words but that interlopers and reports of partial information also repeat. The tendency for TOTs to repeat at a rate greater than can be predicted by chance is consistent with findings in the literature (Warriner and Humphreys, 2008; D’Angelo and Humphreys, 2015). Furthermore, we also replicated the finding previously referred to as the resolution effect, in that TOT states that were self-resolved do not tend to repeat nearly as often as unresolved TOT states do, despite being told the correct answer in all cases. The central finding of this paper is that when using a think aloud protocol, asking speakers to verbalize any information that comes to mind when they are trying to retrieve a word, if a TOT repeats a week later, the same interlopers or partial information often repeats at that time as well; this is over twice as likely for TOTs than Don’t Know Trials. When participants resolve a TOT, they get double exposure to the target word from thinking of the word on their own, in addition to being shown the answer. It is plausible that resolved TOT trials are less likely to repeat than unresolved TOT trials due to the simple explanation of there being more exposures to the target word during a resolved TOT trial. Subsequently, the strengthening of the target word through exposure could make one less likely to repeat a TOT state on Test 2. Although we cannot completely rule out this explanation with these data, one point is that mere exposure cannot explain the fact that TOT states tend to repeat, as Don’t Know Trials do not repeat in the same way as unresolved TOT states, despite the same amount of exposure at Test 1.

These findings demonstrate the importance of the role of phonology in the TOT state. While both Don’t Know and TOT responses in the think aloud protocol frequently contained semantic or episodic information, only the TOT responses showed sizable reports of (usually accurate) phonologically related information. However, this is possibly tautological, in that the presence of consciously available phonological information may make a participant much more likely to describe their state as a TOT rather than a Don’t Know. It is worthwhile to note, however, that available semantic or episodic information does not appear to affect this metacognitive judgment.

The fact that the same information, especially phonologically related material, was reported a week apart on repeated TOT trials speaks directly to our hypothesis about what is being learned in the error state. This finding supports the idea that the TOT state may be best described as an organized pattern of activation, providing an opportunity for convergence on an erroneous state (although as mentioned earlier, this is currently a statistical pattern rather than a systematic one). The retrieval effort, especially a repeated one over a period of 15 s then reinforces the mapping from lemma to that specific incorrect phonological state. Then, during a subsequent retrieval attempt, that incorrectly reinforced mapping is then followed, leading to the same (or at least highly similar) erroneous state, complete with repeated partial information. The learning via Hebbian reinforcement of a specific alternate state also provides a more plausible mechanism underlying an error learning explanation of the error repetition effect. However, we cannot say how this is likely to be the case for all TOT states. It could be common to all, but only occasionally consciously available via introspection, or alternatively may only associate with the subset of cases in which the information is in fact consciously available.

### Limitations and Future Directions

First, there is an alternative possibility that we cannot completely rule out. That is, it is possible speakers repeat partial information not because they are in the same erroneous state during a subsequent recall attempt, but that they are able to explicitly recall the partial information that they were able to come up with a week previously (while still being unable to recall the correct word they had been presented with after their initial TOT, and had verified as their intended target). Future studies should address this possibility.

Second, the numbers reported here tend to be small, as a result of looking at relatively rare error events (which is generally a commonality across all TOT studies), with further diminished numbers by looking at joint probabilities of two of these error events, and furthermore, dividing the classification of the think aloud responses into many different subcategories of type of information. The true rates of each of the subclassifications within recurring errors are therefore difficult to estimate.

## Conclusion

The overall patterns are quite clear: when TOT errors repeat, accompanying partial information can be observed to repeat as well, and TOTs show a much larger proportion of repeated phonological information than Don’t Know responses. To be clear, Don’t Know responses did not necessarily have no information whatsoever; it is possible that participants in a Don’t Know state might have been experiencing a feeling of knowing or deriving semantic information from the definitions. In these cases participants did not know the word and were not in a TOT state as we designated it. In this context, we stress the importance of how differently phonological information is distributed across TOT and Don’t Know responses. We argue that this suggests a fairly organized erroneous pattern of phonological activation within a TOT state, to which a mapping from the lemma level can be reinforced via a Hebbian-type learning mechanism, giving rise to the learning of TOT errors.

## Author Contributions

LO conceptualized the project, collected and analyzed the data, and wrote the Introduction, Materials and Methods, and Results section. KH conceptualized the project, analyzed the data, wrote the Discussion section, and provided edits.

## Conflict of Interest Statement

The authors declare that the research was conducted in the absence of any commercial or financial relationships that could be construed as a potential conflict of interest.

## Appendix

**Table A1:** Lexical and sub-lexical properties of target words.

Target Word	Frequency per Million Words	Orthographic Length	Count of Phonological Neighbors	Mean Concreteness Rating
nectar	2.29	6	9	4.63
audience	76.14	8	1	4.29
dermatologist	2.18	13	3	4.21
gurney	2.11	4	6	4.68
abacus	0.35	6	0	4.52
apron	5.02	5	2	4.87
abdicate	0.37	8	2	2.08
plasma	5.60	6	1	4.17
onomatopoeia	0.09	12	-	-
lava	4.60	4	5	4.82
blubber	0.59	7	2	3.93
glider	1.03	6	5	4.32
subpoena	3.02	8	6	3.46
alchemy	1.31	7	0	2.10
arson	2.50	5	5	3.66
alimony	0.82	7	0	3.08
mausoleum	1.08	9	0	4.23
photosynthesis	1.44	14	0	3.36
origami	0.68	7	0	4.38
gill	4.57	4	40	4.33
farm	55.82	4	10	4.59
spatula	2.43	7	0	4.96
sextant	0.19	7	1	4.30
scapegoat	1.44	9	1	2.52
catalyst	4.71	8	3	2.85
odometer	0.45	8	0	4.72
cartographer	0.23	12	-	3.81
scarab	0.23	6	0	-
regatta	0.46	7	0	-
kaleidoscope	0.99	12	0	4.79
procrastinate	0.29	13	1	2.23
filibuster	2.59	10	0	2.41
ostrich	1.08	7	0	4.71
forfeit	1.42	7	1	2.63
trachea	0.53	7	1	4.48
jettison	0.62	8	1	3.07
shamrock	0.54	8	0	4.89
clavicle	0.38	8	2	4.50
slalom	1.75	6	1	-
perjury	3.68	7	5	2.22
filament	0.86	8	1	4.27
ellipsis	0.22	8	0	3.44
obsidian	0.80	8	0	4.44
contraband	1.73	10	0	3.33
ornithology	0.40	11	0	1.47
spelunker	0.04	9	0	-
incubate	0.25	8	0	3.50
spike	7.68	5	19	4.52
chalice	0.68	7	3	4.83
martyr	2.64	6	15	3.07
gargoyle	0.54	8	1	4.85
station	83.93	7	5	4.32
quarantine	1.98	10	2	3.39
metronome	0.57	9	1	4.27
ventriloquist	0.38	13	-	4.44
trellis	1.00	7	1	-
treason	2.33	7	1	2.03
vineyard	4.45	8	1	4.80
labyrinth	2.86	9	0	4.22
armada	0.84	6	0	4.04
parasite	2.42	8	1	4.34
planetarium	1.70	11	0	4.55
javelin	0.60	7	3	4.90
decanter	0.45	8	1	-
philanthropy	2.62	12	0	2.21
guillotine	0.79	10	0	4.64
allergy	4.01	7	3	3.68
pseudonym	1.21	9	1	2.80
axe	0.52	3	25	5.00
tranquilizer	3.28	12	2	4.55
